# Exploring the Influence of Contextual Factors and the Caregiving Process on Caregiver Burden and Quality of Life Outcomes of Heart Failure (HF) Dyads after a Hospital Discharge: A Mixed-Methods Study

**DOI:** 10.3390/jcm13164797

**Published:** 2024-08-15

**Authors:** Tamara L. Oliver, Breanna Hetland, Myra Schmaderer, Ronald Zolty, Christopher Wichman, Bunny Pozehl

**Affiliations:** 1 College of Nursing, Creighton University, Omaha, NE 68178, USA; 2 College of Nurisng Omaha Campus, University of Nebraska Medical Center, Omaha, NE 68178, USA; breanna.hetland@unmc.edu; 3 Lincoln Campus College of Nursing, University of Nebraska Medical Center, Lincoln, NE 68588, USA; mschmade@unmc.edu; 4 Department of Cardiovascular, University of Nebraska Medical Center, Omaha, NE 68178, USA; ronald.zolty@unmc.edu; 5 Department of Biostatistics, University of Nebraska Medical Center, Omaha, NE 68178, USA; christopher.wichman@unmc.edu; 6 Department of Biostats, University of Nebraska Medical Center, Omaha, NE 68178, USA

**Keywords:** heart failure caregivers, informal caregivers, heart failure dyads, heart failure

## Abstract

**Background**: This study explores heart failure (HF) dyadic contextual factors and caregiver burden during acute exacerbation hospitalization and discharge. **Methods**: It employed a mixed-methods approach, with HF dyads completing questionnaires and semi-structured interviews at a one-week post-discharge outpatient visit. Quantitative tools included the SF-12 Quality of Life, Zarit Burden Interview (ZBI), Bakas Caregiving Outcomes Scale (BCOS), and Self-Care of Heart Failure Index v. 6 (SCHFI). Thematic analysis was conducted on interview data to assess caregiver burden, disease trajectory, comorbidities, caregiving time, and employment status. **Results**: Twelve HF dyads participated, with caregivers (six female, six male) averaging 65.76 years. The ZBI indicated a low caregiver burden (median score of 15), but qualitative data revealed a higher perceived burden related to social isolation, future fears, and caregiver dependence. Male caregivers reported a lower burden than females. Positive goal congruence was noted in caregiving hours and HF management compliance. HF patients had a 10-year survival prediction of 22.75% per the Charlson Comorbidity Index, with 69% in NYHA class III and an average ejection fraction of 37.7%. Caregivers working full-time and caring for higher NYHA-class patients showed higher ZBI and BCOS scores. **Conclusions**: The study highlights the need for mixed methods and longitudinal research to understand HF disease trajectory and caregiver burden, emphasizing the importance of including caregivers in HF education and screening for perceived burden to improve outcomes and reduce re-hospitalizations.

## 1. Introduction

Informal caregivers play an essential role in providing comprehensive and daily management of heart failure (HF) patients’ diet, fluid balance, and medication management to prevent acute exacerbations and hospitalizations [1]. Many patients with heart failure rely on informal caregivers to assist with daily complex HF pharmacological and non-pharmacological regimens to decrease the burden of the disease. Increasing evidence shows that family caregivers improve survival and enhance the quality of life by aiding with activities of daily living, providing direct medical care, monitoring symptoms, and coordinating health services for the patient [1]. While caregiving has been associated with these positive outcomes, it has also been associated with strain and burden [2]. Studies have explored caregiving burden in HF, but a significant gap in the literature relates to the perceived burden of HF caregivers after hospitalization for an acute exacerbation of HF. It is imperative to understand this burden to better prepare and support HF caregivers for the caregiving role after the HF patient is discharged from the hospital [1].

HF family members and significant others often serve in the informal caregiving role, and research indicates these caregivers report that providing care is difficult and overwhelming. Caregivers associate caregiving with increased anxiety, perceived burden, and reduced quality of life [3]. A recent review indicated that HF caregivers reported an elevated level of burden, with an average of 21 h of caregiving per week [1,4]. HF family caregiving burden may include physical, psychological, emotional, social, or financial problems during the time they are caring for an ill family member [5,6]. Previous studies have shown that caregiver burden has been associated with caregivers’ depression and poor quality of life (QOL), as well as patients’ poor outcomes, reduced QOL, rehospitalizations, and death [5,7]. Patient admission to the hospital for an acute exacerbation of HF would be expected to add additional burden to the caregiving role, given many potential changes to the pharmacologic and non-pharmacologic treatment regimen, but this has not been explored in the literature. Understanding the perceived burden from individual caregivers’ unique perspectives immediately following hospitalization for an acute HF exacerbation would provide valuable information to plan supportive interventions. 

**Figure 1:**
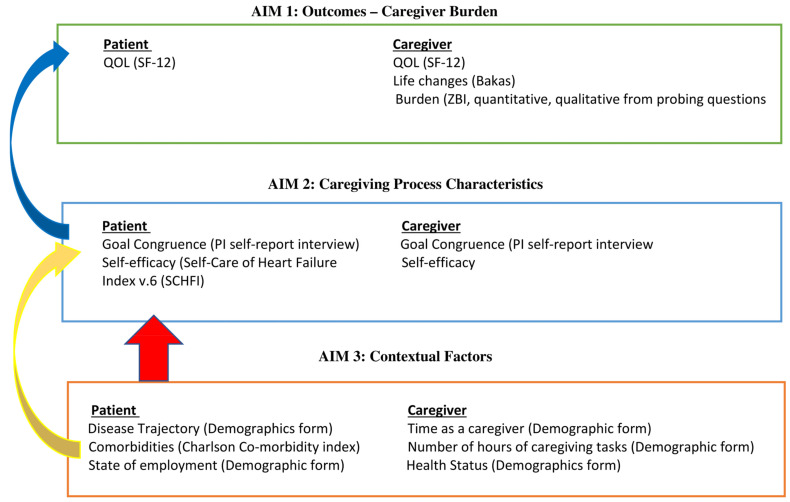
Study Model—adapted individual and family self-management framework [8].

The Individual and Family Self-Management Theory (IFSMT) [8] was chosen as a guide for this study because it considers contextual factors and caregiving process characteristics from both the patient and caregiver perspective that would be particularly important during a time of care transition from hospital to home. This information is important to consider when evaluating caregiving outcomes in terms of burden for the caregiver and quality of life for both the patient and caregiver [8]. IFSMT proposes that condition-specific risk and protective factors (physical and social environment, and characteristics of the caregivers or family members) may directly impact the proximal outcomes of the HF patient [8]. The adapted model for this study (Figure 1) describes contextual factors from both the patient (disease trajectory, comorbidities, NYHA, ejection fraction) and the caregiver (time as a caregiver, number of hours of caregiving per week, employment status, and health status) that may impact caregiving burden and quality of life outcomes [9]. Caregiving process characteristics (self-efficacy care reported by patient and caregiver) and goal congruence between patient and caregiver will be described and related to caregiving burden and quality of life for both patient and caregiver. See Figure 1 for the study model.

## 2. Purpose

The purpose of this study was to explore HF caregiver burden and describe contextual factors and caregiving process characteristics on proximal outcomes of burden for the caregiver and quality of life for both patient and caregiver. The study aims to: 

Aim 1: Describe patient and caregiver outcomes at the one-week post-hospital follow-up visit—caregiver burden (quantitative ZBI and qualitative interview); Bakas Caregiving Outcomes Scale (life changes); quality of life from both caregiver and patient. 

Aim 2: Describe caregiving process characteristics, including caregiver and patient self-efficacy (self-report) and goal congruence (semi-structured interview). 

Aim 3: Describe patient and caregiver contextual factors that may affect the dyadic caregiving process and outcomes. Patient contextual factors include demographics, disease trajectory, comorbidities, NYHA, and ejection fraction at the time of hospitalization. Caregiver contextual factors include time as a caregiver, number of hours per week of caregiving, employment status, and current health status. 

## 3. Methods

### 3.1. Design

A cross-sectional convergent mixed-methods design was used. Data collection methods included self-reported demographics, surveys, medical record extractions, and semi-structured interview data collected from the dyad. Data collection occurred between February and July 2022.

### 3.2. Ethics Approval

The Institutional Review Board approved the study prior to recruitment or data collection occurring (IRB#0037-22-EP). Participant consent was obtained from each eligible participant (both the HF patient and caregiver signed separate consents for the study) prior to data collection. Study data were collected via paper surveys, a paper demographics form, and recorded interviews that were transcribed verbatim. The data were entered and stored in a password-protected Excel document. 

### 3.3. Sample

A convenience sample (n = 12) of HF dyads (patient and caregiver) was recruited from the Heart Failure inpatient setting or the Heart Failure outpatient clinic located in the midwestern United States. To be eligible to participate, HF dyads needed to meet the following criteria independently. Caregiver inclusion criteria: (a) caregiver of an HF patient within one week of an inpatient hospitalization for an acute exacerbation of HF; (b) English-speaking only; (c) competent to consent on their own behalf. Patient inclusion criteria: (a) patient with previously diagnosed HF and admitted for an acute exacerbation of HF; (b) English-speaking only; (c) has a dedicated caregiver that lives with them or spends five days managing the patient’s care; (d) is competent to consent on their own behalf. Patients and caregivers were excluded if the patient was being discharged to a skilled nursing home (short- or long-term care). 

### 3.4. Recruitment, Enrollment, and Data Collection

HF dyads were recruited from the HF inpatient service or the HF outpatient clinic with the assistance of the heart failure team, which consisted of nurse practitioners, heart failure physicians, and heart failure nurse navigators. The heart failure team presented the study opportunity to the eligible HF dyads to gain permission for the principal investigators to contact the HF dyads for this research study. HF dyads were consented to in person, either in the inpatient setting or at the one-week outpatient follow-up discharge visit. At the one-week post-discharge visit, the HF patient and caregiver completed the assigned questionnaire packets and the interview separately with the PI in a private room. Dyads completing all data collection received a $25 Amazon card. 

### 3.5. Individual and Family Self-Management Theory (IFSMT)

The Individual and Family Self-Management Theory (IFSMT) being utilized in this study to educate heart failure caregivers at the time of discharge is justified for several reasons. The IFSMT offers a comprehensive framework that emphasizes the interplay between individual and family contexts, the process of self-management, and the outcomes of self-management. IFSMT considers both individual and family factors, making it particularly suited for heart failure caregivers who often operate within a family context. This holistic view helps address the multifaceted needs of both patients and caregivers. The theory emphasizes the importance of contextual factors such as personal, environmental, and social influences. In heart failure caregiver literature, certain contextual factors are often not adequately studied or controlled, potentially confounding the outcomes for both patients and caregivers. These factors can influence the caregiving process and the effectiveness of interventions, leading to a skewed or incomplete understanding of the caregiving experience [1]. Caregiving process characteristics encompass various aspects that define the dynamics and quality of the caregiving experience. In this context, they specifically include the self-efficacy of both the patient and the caregiver. The caregiving process in the context of heart failure involves a series of activities and interactions that caregivers undertake to manage the care of heart failure patients. This process is multifaceted and includes various tasks, emotional support, and coordination with healthcare services [1]. At discharge, understanding these factors can help tailor education and support to the specific circumstances of each caregiver and patient. Educating caregivers using this framework ensures they are equipped with the necessary skills and support systems to manage heart failure effectively. By using IFSMT, education programs can aim for improved health outcomes, quality of life, and caregiver satisfaction. Using IFSMT to guide education for heart failure caregivers ensures a structured, comprehensive approach that addresses the complex needs of both patients and their caregivers, ultimately leading to better management of the condition and improved outcomes.

### 3.6. Dyad (Patient and Caregiver) Measures

Demographics and Contextual Factors. The patient and caregiver completed a demographics form to provide contextual characteristics, including age, employment status, and education level. 

Caregiving Process Characteristics. Caregiving process characteristics included self-efficacy for both the patient and caregiver. The Self-Confidence subscale of Self-Care of Heart Failure Index v.6 (SCHFI) was used to assess self-efficacy in the patient. Caregiver self-efficacy was measured with the General Self-Efficacy scale.

Self-efficacy: Self-Care of Heart Failure Index v.6 (SCHFI) for the HF Patient. The SCHFI v.6 has 22 items and consists of three scales measuring self-care maintenance, self-care management, and self-care confidence. For this study, we used the self-care confidence scale to measure HF self-efficacy. The SCHFI uses a 5-point Likert-type choice and is anchored from 1 “not confident” to 5 “extremely confident”. The instrument was developed for heart failure patients and is reported as reliable and valid [10]. The scale will be administered to all patients who consent to the study. The possible range for the Self-Confidence subscale of the SCHFI v.6 is 5–30, with higher scores indicating better self-care confidence in the ability to perform self-care behaviors by HF patients. The reliability of SCHFI v.6 was shown to be 0.70 [10].

General Self-Efficacy for the caregiver. The General Self-Efficacy (GSE) scale is a 10-item scale that is designed to assess optimistic self-beliefs to cope with the demands of life [11]. The four-point Likert scale is anchored from 1 “not true at all”, to 4 “exactly true” on the questions. Higher scores indicate better self-efficacy (total score between 10 and 40). The scale has been found to be dependable and valid in chronic disease caregivers [11] and is positively correlated with motion, optimism, and work satisfaction. It is negatively correlated with depression, stress, health complaints, burnout, and anxiety. The reliability of the GSE is between 0.76 and 0.90 [11].

Goal Congruence—Dyad Semi-Structured Interviews. Separate interviews with the HF patient and caregiver, using an investigator-developed semi-structured interview, were completed. See the interview questions in Appendix A. Dyad congruence was assessed by asking the patient and caregiver seven questions about compliance with diet, medications, exercise, and heart failure symptom management. Interviews were audio-recorded and transcribed verbatim. Answers were descriptively compared with determine congruence between the patient and caregiver concerning goals of care. 

SF-12 Quality of Life (QOL). The Short Form Health Status Questionnaire-12 (SF-12) is a generic instrument used to assess quality of life [12]. SF-12 scores range from 0 to 100, with higher scores meaning better quality of life. Two subscales compose the SF-12: a physical QOL and a mental QOL. Each subscale (physical and mental) ranges from 0–100, with high scores corresponding to a better quality of life. Self-reported quality of life will be evaluated by both the patient and caregiver. The reliability of the SF-12 has been shown to be 0.890 [12].

### 3.7. Measures for Caregiver Only

The Zarit Burden Interview (ZBI). The ZBI was originally developed to assess burden among caregivers of community-dwelling dementia patients [13]. It is the most commonly used instrument to assess caregiving burden in clinical and research settings [5] and has been used to assess burden among caregivers of HF patients [14]. The ZBI is a 22-item instrument that uses a 5-point Likert scale with responses of 0 (never) to 4 (nearly always). The sum of scores across the 22 items ranges from 0 to 88, and a score of 17 or higher indicates high caregiver burden. The dimensions reported to be measured by the ZBI specific to HF caregivers include the consequences of the caregiver, the patient’s dependence, exhaustion and uncertainty, and guilt and fear for the patient’s future. Cronbach’s α for the ZBI in HF was 0.921 [5].

ZBI Qualitative Interview—Semi-Structured Probing Interview. The semi-structured interview to obtain qualitative data involved additional probing questions for any items of the ZBI that were answered with “quite frequent” (rating of 3) or “nearly always” (rating of 4) present. HF caregivers were asked to re-read any question that was answered with a 3—“quite frequently”, or 4—“nearly always” answer and asked to explain further. Probing questions involved asking the caregiver to “explain further” or “give me an example”.

Bakas Caregiving Outcomes Scale (BCOS). The BCOS is a 19-item instrument designed to measure life changes resulting from caregiving related to emotional well-being, ability to cope with stress, self-esteem, relationships with friends and family, physical health, time for social activities, future outlook, and relationship with the care recipient [15]. The scale has 19 items on a 7-point Likert scale from −3 (worst changes) to +3 (changed for the best). The −3 to +3 ratings are recoded to scores of +1 to +7, so that positive numbers can be obtained for analysis. Scores > +4 indicate that the caregiver’s life has changed for the better, whereas scores ≤ +4 indicate negative perceptions of the caregiving experience [15]. Bakas [15] indicated that the instrument was originally developed to measure life changes in family caregivers of stroke survivors but has been used for heart failure and other chronic conditions. Cronbach’s α was 0.90 [15].

### 3.8. Measures for HF Patient Only

Charlson Comorbidity Index (CCI). The Charlson Comorbidity Index (CCI) was developed in 1987 as a weighted index to predict the risk of death for hospitalized patients [16,17]. The index includes 19 conditions, with each assigned a weight from 1 to 6, based on the estimated one-year mortality hazard ratio and age: ages 55–60 are two points; 61 to 70 are three points; and ages 71 and older are four points. A single point is given for each of the following conditions: previous myocardial infarction, congestive heart failure, peripheral vascular disease, cerebrovascular disease, dementia, chronic pulmonary disease, hypertension, connective tissue disease, mild liver disease, and diabetes. Conditions receiving two points would include hemiplegia or paraplegia, moderate or severe renal disease, diabetes with chronic complications, cancer without metastasis, leukemia, and lymphoma. Moderate or severe liver disease, cancer with metastasis, and HIV/AIDS would receive three points. HF patients are a noted population with several comorbidities, including chronic renal failure, cerebrovascular accidents, chronic obstructive pulmonary disease, stroke, and dementia [18]. Co-morbidities were collected from the patient and caregiver by self-report.

New York Heart Association (NYHA). The NYHA was collected on each patient via medical record review. The New York Heart Association (NYHA) classifies patients’ heart failure according to the severity of their symptoms and how limited they are during physical activity [19]. See Table 1.

### 3.9. Data Analysis

A sample size of 30 was planned, based on previous intervention studies, to achieve correlation between dyad groups. However, due to the COVID-19 pandemic, the institution shut down visitation during January and April 2022. Caregivers were not allowed into the hospital or outpatient setting, and the original sample size was not obtainable in the allocated timeframe for this study. Saturation was achieved for the qualitative data from the semi-structured interview with the 12 dyads. Descriptive statistics were summarized for each aim rather than performing inferential statistical tests. 

Aim 1: Describe patient and caregiver outcomes—caregiver burden (quantitative ZBI and qualitative interview), Bakas Caregiving Outcomes Scale (life changes), quality of life (both patient and caregiver), collected at the one-week post-hospital follow-up visit. The first phase was to collect the quantitative scores from the ZBI to identify questions the caregiver answered with a 3 “quite frequently” or 4 “nearly always”, indicating an increased level of burden. The second phase, with the semi-structured interview, involved asking probing questions to provide more in-depth information concerning the perceived burden associated with those questions answered with “quite frequent” (rating of 3) or “nearly always” (rating of 4) present. HF caregivers were asked to re-read any question that was answered with a 3—“quite frequently” or 4—“nearly always” answer and asked to explain further. Probing questions involved asking the caregiver to “explain further” or “give me an example”. Data from the ZBI were scored with the median and interquartile range for each caregiver. Semi-structured interviews were conducted to obtain additional information concerning perceived burden. Interviews were recorded and transcribed verbatim. Thematic analysis was completed by two authors to confirm themes (TB, BP) on all the transcripts from the results of the probing questions. The same interviewer (TB) conducted all the interviews and read from the same interview guide (Table 2) to ensure the reliability of the data. The data were coded by the principal investigator and confirmed by the second author (BP) for consistency. Descriptive statistics (frequency, mean, standard deviation, median, and interquartile range) were used to describe the Bakas Caregiving Outcomes Scale for the caregiver and the quality of life measured in both the patient and the caregiver.

Aim 2: Describe caregiving process characteristics, including caregiver self-efficacy and dyadic goal congruence. Data were analyzed and reported descriptively (frequency, mean, standard deviation, median, and interquartile range) for self-efficacy for self-care (patient) and self-efficacy for caregiving (caregiver). Responses to the goal congruence semi-structured interview were transcribed and compared between the caregiver and patient to determine, with a second author, if the dyad exhibited congruence or incongruence in the goals of care (see Table 3). The number of hours of caregiving reported by patients and caregivers was evaluated. 

Aim 3: Describe patient and caregiver contextual factors that may affect the dyadic caregiving process and outcomes. Patient contextual factors include disease trajectory, comorbidities, NYHA, and ejection fraction at the time of hospitalization. Caregiver contextual factors include time as a caregiver, employment status, and current health status. Descriptive statistics (frequency, mean, standard deviation, median, and interquartile range) were summarized for each of the patient and caregiver contextual factors.

## 4. Results

### 4.1. Demographic Analysis

A sample of 12 HF dyads was recruited and participated in this study. The caregivers consisted of six females and six males, with a mean age of 65.76 years (SD = 13.48). Heart failure patients consisted of six males and six females with a mean age of 64.77 years (SD = 14.56). Dyads were predominantly Caucasian, with only one African American dyad. A complete description of the sample demographics can be found in Table 2.

**Figure 2:**
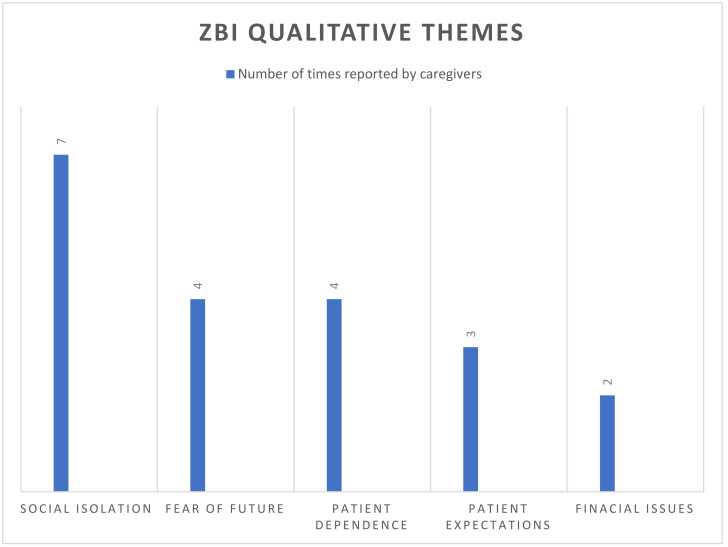
ZBI qualitative themes—frequency of response by theme.

Aim 1: ZBI scores among the caregivers showed a median of 15 and an interquartile range of 4–31. A total of 16 questions in the survey were answered “quite frequently” (18 times) or “nearly always” (8 times), which prompted the probing questions among the 12 caregivers (see Table 4). Codes were derived from the transcriptions of the qualitative interview and organized into themes by the PI and two co-authors. Themes were derived using the iterative process, relying on subjective analysis of the caregivers responses to identify recurring themes such as social isolation from COVID-19 and fear of the future due to HF. Once themes were agreed upon, the PI coded the remaining data and confirmed it with the other two co-authors. The analysis from the ZBI interviews produced five main themes among caregivers, which included social isolation, fear, patient dependence on caregivers, patient expectations, and financial strain. Figure 2 reports the frequency of responses to each of the five themes. Qualitative findings from the ZBI semi-structured interview, with excerpts of probing questions and themes, are summarized below in Figure 2. 

#### 4.1.1. Social Isolation

Although the ZBI does not include questions specifically related to the COVID-19 pandemic, this study was completed during the active pandemic. Over half of the caregivers commented on social isolation during the interview. Lack of support was reported by four of the caregivers, especially during the pandemic. Six of the caregivers stated that during the pandemic, they were not allowed to go to the hospital for a face-to-face discharge instruction with the nurse and HF coordinator, which created barriers and confusion. One caregiver indicated she felt “invisible” to the care team when not allowed to be present at hospital discharge or for outpatient visits due to the COVID-19 virus.

The following quote from one of the caregivers illustrates COVID-19 social isolation: “*My social life has been a struggle with family and friends because of COVID. I want to keep him safe without the risk, so I keep company at a minimum and require everyone to wear a mask and be vaccinated to enter the house*.”

#### 4.1.2. Fear

The caregivers exhibited several accounts of fear regarding the HF patient throughout the interviews. For example, the ex-spouse was incredibly worried about the future of the patient due to his non-compliance with the doctors’ orders and very low ejection fraction. Three of the spouses reported they were fearful of losing their spouses to the HF disease and wanted to try to do everything and listen to physicians’ orders to keep their spouses alive and well. Another fear, reported by two of the caregivers, was that when the patient was in the hospital, they feared bad news and receiving a call that the patient’s condition had worsened. Three caregivers reported that the trajectory of the HF disease was a challenge because of the unknown future of the patient and their fear of the future. Finally, one of the caregivers had lost her parents to heart disease and heart failure and indicated that this created an ongoing fear.

An example of fear is illustrated in this quote from one of the caregivers:

“*His last hospital stay really scared me, and I was not really sure or got an answer about his prognosis*.”

#### 4.1.3. Patient Dependence upon Caregivers

Caregivers reported continued patient dependence on the caregiver in both the ZBI and during the interview. Three of the twelve caregivers reported that the patient had daily “expectations” from them and answered Question 8, “Does your relative expect you to take care of him/her, as if you were the only one he/she could depend on?” with a 3 or 4 on the ZBI. Caregivers expressed that they felt the patient could do some of the tasks but “allowed” the caregiver to because the patient was a spouse or parent.

An example quotes one of the caregivers: “*I am the only one that is even available. My first career was as a CNA, and I took care of his sister before she died. Both sets of parents passed away, and honestly, I am the only one. He would do it for me. We have been married for 25 years*.”

#### 4.1.4. Patient Expectations

Four of the caregivers answered question 14, “Your relative seems to expect you to take care of him/her as if you were the only one they could depend on”, with a 3 or a 4. The caregivers reported that they were the only people available to take care of the patient, either as their parents or significant other. Two of the caregivers had been caregivers in the past for previous family members.

An example quotes one of the caregivers: “*Yes. He defers to me even though he enjoys some of the tasks with other family or friends, such as going to Menards. I think he would enjoy these much better if he went with his friends or male relatives, but he wants me to go in case something happens*.”

#### 4.1.5. Financial Strain

Question 15 asked the question, “Do you feel that you don’t have enough money to take care of your relatives in addition to the rest of your expenses?" Four out of the twelve caregivers indicated that they “nearly always” felt they didn’t have enough money. Caregivers indicated that the patient was not able to work, and the income depended upon them, as well as the burden of caregiving. One of the caregivers indicated that there was discussion of a possible heart transplant, and he had recently met with the financial counselor. The “price tag” for the heart transplant scared him, as he and the patient relied solely on the caregiver’s employment income. He was also concerned about the addition of post-transplant caregiving commitments for the patient. The caregiver, who was a parent, indicated she had to retire to take care of her daughter and continues to be on a fixed income, and she felt a bit guilty not being able to afford to buy a car or nice things for her 23-year-old daughter. For each of the four caregivers reporting financial strain, the caregiver was the only source of income for the dyad, and this was a source of burden among these four caregivers.

A quote from one of the caregivers illustrates perceived financial strain and potential burden: “*Yes, she has not worked since 2016 and she has been in the hospital with so many visit(s) and now they have thrown around working her up for heart transplant and I am concerned with the cost of the surgery, hospital and post-transplant medications what our out of pocket and monthly costs are for us as I am the only income and we have a son still at home*.”

Quality of life was measured by the SF-12 (physical and mental subscales) for the dyads (Table 5). Caregivers reported a mean of 47.52 (SD = 7.67) on the physical subscale and 52.80 (SD = 8.46) on the mental subscale. HF patients reported a mean of 32.24 (SD = 8.87) on the physical subscale and 53.85 (SD = 8.46) on the mental subscale.

Quality of life scores for the patient’s SF-12 physical subscale score were relatively low (mean of 32.24 SD = 8.87), indicating less ability to complete physical tasks. However, the mental score was just slightly above average, with a mean of 53.85 (SD = 10.96).

Life changes were measured for the caregivers through the Bakas Caregiving Outcomes Scale. The mean score of the caregivers was −2.72 (SD = 6.63), indicating a score well below the cut-off of a positive 4. This indicates that negative changes have taken place in the caregiver’s life since discharge.

Aim 2: Describe caregiving process characteristics, including dyad self-efficacy and goal congruence. Goal congruence will be assessed through interviews (qualitative) with the dyad.

Self-Efficacy. HF patient SCHIFI scores for the self-confidence subscale had a mean of 14.83 (SD + 4.20), indicating lower self-care efficacy [10]. Caregivers scored a mean of 29.75 (SD = 8.38) on the General Efficacy Scale.

**Figure 3:**
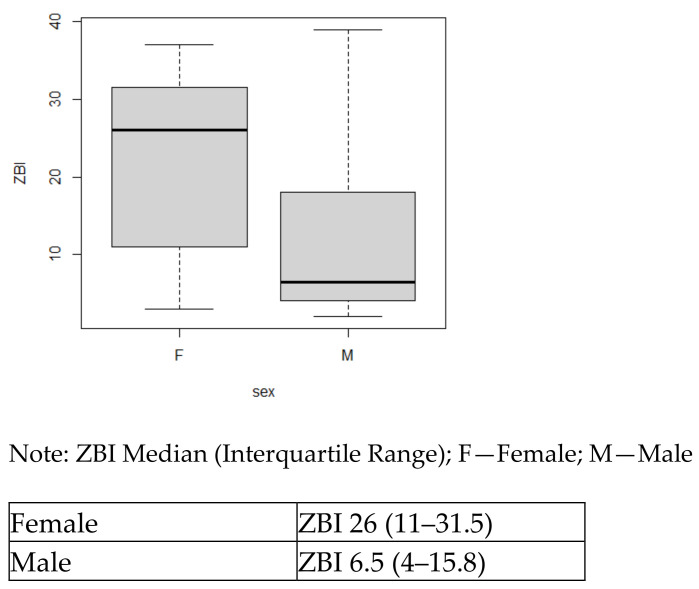
Boxplot for ZBI by sex.

Dyad goal congruence. Dyad congruence was captured using seven interview questions that were asked of each part of the dyad and compared (Table 3). The average number of hours reported by caregivers per week was 6.38 h, versus 5.54 h reported by HF patients. A Bland Altmann analysis (Figure 3) showed that the difference in caregiving time between caregivers and HF patients was within the limits of agreement (92.3% (66.7–98.6%)) for the 12 dyads. HF goals for patient and caregiver were similar in that over half of the dyads reported the number one goal was to stay out of the hospital. Remaining results indicated caregivers’ goals were task-oriented, such as completing daily prescribed exercises (physical therapy), medication adherence, daily blood pressure and weights, and a low sodium diet. HF patient goals focused on staying out of the hospital (seven answered), daily exercise regimen (three), taking medications as prescribed (two), and managing daily symptoms (two). Goal congruence was evaluated by an independent review of responses to the questions and answers by two of the authors. Conflicting reviews were resolved through discussion by the two authors.

Aim 3. Describe patient and caregiver contextual factors that may affect the dyadic caregiving process and outcomes. Male caregivers reported lower burden scores (ZBI) with a mean of 12.7 (SD = 12.91), compared with female caregivers with a mean of 21.6 (SD = 10.43). However, the median and interquartile range offer a better look at these data, showing a female median of 26 (IQR 11–31.5) and a male median ZBI score of 6.5 (IQR 4–15.8), indicating a larger difference between male and female burden scores.

Two of the three caregivers of patients with NYHA Class IV HF reported the most negative life changes, with a −13 and −12 score on the BCOS. Additionally, caregivers with higher burden and ZBI scores of 31, 37, and 39 were providing care for the patients with NYHA IV HF. There was no clear influence of the age of the caregiver on the perceived burden. Relationship status among the dyads showed that 67% of them reported being married, with qualitative data indicating some couples having been married for many years. The relationship of the caregiver to the patient also showed no influence on burden.

Table 2 summarizes descriptive statistics of all the dyad demographics that were contextual factors evaluated in relation to burden.

## 5. Discussion

This study explored patient and caregiver outcomes and described the specific caregiving process characteristics and contextual factors that may affect caregiver burden after a recent HF patient discharge. HF dyads may be more susceptible to a perceived increase in caregiver burden at the one-week discharge follow-up visit from a recent acute HF exacerbation. To our knowledge, this is the first study to explore contextual factors and caregiving process characteristics’ effect on outcomes of the HF dyads utilizing burden tools, QOL, and interviews. The study used a mixed-methods design that provided more context and process characteristics related to caregiving burden. Quantitative measures may only capture scores to prove the broad general points of burden, whereas qualitative interviews may bring richer details and the depth to understand the full implications of HF caregiver burden [20] at the post-discharge one-week clinic visit after a recent HF admission.

While the literature describes the perceived burden for caregivers of stable chronic patients with HF, there has been limited research to examine the perceived caregiver burden following a hospitalization for an acute HF exacerbation [3]. Caregiver burden reported by the quantitative ZBI revealed a median score of 15, indicating low burden for this group of caregivers. Recent studies have shown much higher ZBI scores of 19.43 to 37.7 in stable chronic heart failure patients [21,22]. Our findings from the quantitative ZBI results indicating low caregiver burden were unexpected, given the recent hospitalization for an acute exacerbation of HF. Reasons for the potentially low caregiver burden score discrepancy may include experienced caregivers, caregiver resilience, role acceptance, and the timing at which the ZBI was administered compared with the actual burden. Changes in clinical outcomes, such as hospitalization for an acute exacerbation, have been shown to increase caregiver burden [23]. Additionally, the Bakas Caregiving Outcomes Scale, measuring life changes in family caregivers of persons with a chronic illness, showed a significant negative number (−2.72) in this sample, indicating negative perceptions of the caregiving experience by these caregivers following the patients’ hospitalization [15]. These findings from the Bakas Caregiving Outcomes Scale would suggest that these caregivers were experiencing burden that was not detected with the ZBI. The use of the ZBI to quantify caregiver burden in HF may not be sensitive to changes in caregiving burden that result from a recent hospitalization for an acute exacerbation of HF. The Bakas Caregiving Outcomes may be more sensitive to capturing changes in caregiving burden that result from challenges experienced in relation to the transition from hospital to home for patients with HF. The qualitative findings from the semi-structured interview also support that these caregivers were experiencing burden that was not detected in the quantitative ZBI measure, but the semi-structured interviews provided specifics of actual burden. The qualitative findings are important and would be invaluable when planning interventions to support caregivers of patients with HF.

Patient quality of life scores (SF-12) on the physical subscales were relatively low (mean of 32.24, SD = 8.87), indicating less ability to complete physical tasks. However, the mental score was just slightly above average, with a mean of 53.85 (SD = 10.96). These results are not surprising, given the recent hospitalization for worsening HF and the physical limitations that surround HF patients, such as shortness of breath and fatigue. Lack of a caregiver in heart failure patients has also been associated with increased risk of death and decreased quality of care or quality of life for the patient [1]. Additional research, especially measuring quality of life over time, is needed to determine the effects of the hospitalization on the patient’s quality of life.

Caregiver quality-of-life outcomes are also important to consider. Caregivers SF-12 physical subscale scores were also lower than average (<50), with a mean of 47.51 (SD = 7.67), and just above average on the mental subscale, with a mean of 52.8 (SD = 8.46). Heart failure caregivers who experience burden are at risk for significantly higher physical and psychological health risks, and the current literature has less scientific insight into their specific needs and outcomes [24]. It is also important to note that HF caregiver burden has been shown to have a negative impact on patient outcomes, resulting in decreased and worsening physical function and more mental health issues for the patient [2]. Caregiver burden impacts patient and caregiver outcomes and is multi-faceted. Research is needed to explore and understand all aspects of burden, especially at a time of hospitalization for an acute exacerbation of HF [25].

### 5.1. Caregiving Process

The experience of HF can be explained as a shared one by both the patient and caregiver [1]. Caregivers play an essential role in providing physical, practical, and emotional care to HF patients that changes over the trajectory of the disease [1]. Several studies have reported that social support from family caregivers is associated with better HF outcomes for the patient, such as lower hospital readmissions and medication compliance [1]. The caregiving process was measured in this study by self-efficacy and goal congruence for the dyad. The patient completed the self-confidence subscale of the Self-Care of Heart Failure Index v.6 (SCHFI) to measure self-efficacy. The mean score of the patients was 14.83 (SD = 4.20), with a possible highest score of 30 for this subscale, indicating these patients were not feeling confident in their ability to engage in self-care behavior [10]. Lower self-efficacy scores have been attributed to higher HF caregiver burden and strain [10]. Caregiver self-efficacy was better than average, with a score of 29.75 (SD + 8.38), and could indicate why there may have been less perceived caregiver burden on the quantitative ZBI.

#### Dyad Congruence

Roles and interactions within this dyadic relationship are interdependent and may show congruence or incongruence. Congruence can be defined as consistent perceptions about the HF illness between the dyad [26]. Incongruence may be described as the difference between the dyad’s decisions, attitudes, and behaviors [1]. Incongruence between the HF dyad has been shown to affect illness management and potentially add increased burden to the caregiver [1]. Incongruence can also be attributed to a lack of communication between the dyads. Congruence was measured using the same semi-structured interview (seven questions) in a separate interview with the patient and caregiver. Staying out of the hospital for the patient was the number one goal, followed by adherence to medications. Caregiver goals were congruent and included staying out of the hospital, symptom management, and low-sodium diet goals. All the dyads were congruent in the “compliance” questions (medication adherence, exercise, diet, and daily symptom management), indicating overall agreement or congruence in questions related to compliance with the treatment regime. Our findings of dyadic congruence between caregiver and patient indicate that hospitalization and transition to home did not result in incongruence, which could increase perceived burden. Dyadic congruence has not been explored in the HF literature, and these findings would need to be validated in a larger, more diverse sample.

### 5.2. Contextual Factors

Caregiver characteristics (contextual factors), such as older age, female sex, and the number of hours of caregiving per day, may predict a higher burden [1]. Our findings show caregivers reporting fewer hours of caregiving (mean 6), as compared with a recent review reporting an average of 21 hours of caregiving per week [1,4]. This could potentially be explained by the stage of HF and the severity of the illness. Only 25% of our sample were NYHA Class IV, who require more care and assistance. Our findings are consistent with previous literature reporting females having a higher number of caregiving hours and increased burden in comparison to their male counterparts [27]. Most trials report predominantly female caregivers, whereas our study had equal numbers of female and male caregivers. Male caregivers had significantly lower burden scores (mean 10.83) than female caregivers (mean 24.67) in our study. The general caregiving literature reports less burden for male caregivers, in part because men are more frequently offered and accepting of help from others as compared with women [28]. Currently, there is not any data to support this conclusively with HF caregivers.

A recent review indicated that most HF caregivers report a high level of burden, especially those who are older caregivers and have multiple comorbidities [4]. Additionally, burden has been related to caregivers who take care of patients with lower mental quality of life and an increased trajectory of disease (NHYA III and IV) (Durante et al., 2019 [27]). Hu [21] performed a similar study during hospital transition in China with much higher ZBI scores (mean 37.1) among younger caregivers (children of HF patients) and attributed the higher burden to financial burden and insufficient resources. Our study only had one younger caregiver who was a child of the HF patient, which may in part explain the lower level of reported caregiver burden. Further study to examine the burden on younger children in the caregiver role is needed.

Contextual factors that influenced burden scores included caregivers who worked full-time jobs and took care of those patients with a higher trajectory of disease (NYHA = IV). Caregivers who worked full-time and were the primary caregiver did have higher ZBI burden scores and BCOS (life changes) scores. Length of time as a caregiver did not influence a higher burden or affect the caregiving process. Average hours of caregiving have been shown to increase with the NYHA class of III–IV by up to eight hours a week [29]. The current sample had three NYHA class IV patients and was consistent with Lahoz [29], with higher burden scores in caregivers of NYHA class III as compared with NYHA class II patients. NYHA IV classifications include individuals who are not able to carry out any physical activity without significant symptoms. Therefore, caregivers would be expected to provide more assistance with daily activities.

The severity of the patients’ condition at discharge is a pivotal factor influencing the endpoints studied. Patients with severe heart failure at discharge require more intensive care, which can exacerbate caregiver burden and emotional distress. This increased demand can strain caregivers’ physical and emotional resources, leading to a decline in their quality of life. Understanding this dynamic is crucial for developing effective interventions that address the specific needs of caregivers based on the severity of the patients’ condition. Further research should explore targeted strategies to mitigate the impact of high patient severity on caregivers, ensuring comprehensive support mechanisms are in place.

### 5.3. Limitations

The results of this study are limited by the cross-sectional design and collection of caregiver burden at one point in time. Findings are also limited by the small sample and primarily spousal dyadic relationships. Originally, a sample size of 30 was planned, but due to an institutional restriction on visitors during the COVID-19 pandemic, we were unable to recruit dyads (both patients and caregivers) for participation in this study. In addition, the sample was recruited from one institution, resulting in limited diversity and consisting predominantly of Caucasian HF dyads. Recruiting from a specific geographical area might lead to a sample that lacks ethnic and racial diversity, affecting the generalizability of the findings to broader populations. Cultural differences in health beliefs and behaviors can affect how heart failure is perceived, treated, and managed. All data were self-reported by the dyads, apart from the NYHA and ejection fraction, which may be biased by the social desirability of responses. Future research should include a larger, more diverse sample with different dyadic relationships between patient and caregiver (children, parents, non-living caregivers, and siblings as caregivers).

## 6. Conclusions

This cross-sectional, mixed-methods study is one of the first designed to explore caregiver burden following a patient’s recent hospitalization for an acute exacerbation of HF. Qualitative semi-structured interviews, the effects of contextual factors, and caregiving process characteristics provided important information concerning perceived burden. Caregiver burden, quantified with the ZBI, showed burden to be low; however, qualitative data and the Bakas Caregiving Outcomes Scale indicated burden that was not identified by the ZBI. Specific contextual factors that impacted caregiver burden included age, employment status, hours of caregiving, symptom burden, or the NYHA classification of the HF patient. Older caregivers who work full-time need to be screened for increased burden, especially if the HF patient has a higher NYHA classification and more symptoms. HF dyads reported lower QOL scores in both the physical and mental scoring of the SF-12. This has not been previously explored following a recent hospitalization and would benefit from a study of longitudinal effects over the HF trajectory, which often includes multiple hospitalizations. The ZBI failed to capture the true burden of these HF caregivers following the hospitalization of the patient for an acute exacerbation of HF. The BCOS may be an important screening measure to evaluate the perceived burden of caregivers following a HF hospitalization. An increased burden may have important implications for discharge planning following hospitalization. Nurse navigators to assist with discharge planning could assess caregiver burden and help find appropriate support for unmet needs. Further research is needed to develop and test interventions to reduce caregiver burden following hospitalization of the patient with HF.

In conclusion, this study investigated the impact of contextual factors on heart failure caregivers’ outcomes. Our findings suggest that caregiver burden, emotional distress, and quality of life are significantly influenced by various contextual elements such as social support, caregiver–patient relationship quality, and caregivers’ health status. Importantly, the severity of the patient’s condition at discharge plays a critical role in shaping these outcomes. Specifically, higher severity at discharge was associated with increased caregiver burden and emotional distress, underscoring the need for tailored support interventions. These insights highlight the necessity for healthcare providers to consider these factors in post-discharge planning and caregiver support programs.

## Figures and Tables

**Table 1:** New York Heart Association.

Class	Patient Symptoms
I	No limitation of physical activity. Ordinary physical activity does not cause undue fatigue, palpitation, or dyspnea (shortness of breath).
II	Slight limitation of physical activity. Comfortable at rest. Ordinary physical activity results in fatigue, palpitation, or dyspnea (shortness of breath).
III	Marked limitation of physical activity. Comfortable at rest. Less than ordinary activity causes fatigue, palpitation, or dyspnea.
IV	Unable to carry on any physical activity without discomfort. Symptoms of heart failure at rest. If any physical activity is undertaken, discomfort increases.

Ejection Fraction %. The ejection fraction was collected via the patient’s electronic medical record. Ejection fraction measures the ability of a person’s heart to pump effectively. Normal ranges for ejection fraction are 50% to 70%.

**Table 2:** Demographics of sample (n = 12).

Characteristic	HF Patient Frequency (%)	Caregiver Frequency (%)
Age	65.76 years (SD = 13.48)	64.77 years (SD = 14.56)
Relationship status	Married = 8 Significant other = 2 Child of HF patient = 1 Mother of HF patient = 1	
Sex		
Male	6 (50%)	6 (50%)
Female	6 (50%)	6 (50%)
Race		
White	11 (91%)	11 (91%)
African American	1 (9%)	1 (9%)
Employment status		
Working full-time	0	3 (25%)
Working part-time	0	1 (8%)
Retired	12 (100%)	8 (67%)
Highest education level		
Less than high school	1 (8%)	0
Graduated high school	10 (83%)	5 (42%)
Some college	1 (8%)	4 (33%)
College graduate	0	3 (25%)
Hours of caregiving per week		
Hours reported by patient	5.54	
Hours reported by caregiver		6.18
Cardiovascular risk factors	Hypertension Diabetes Mellitus II Atrial Fibrillation	Hyperlipidemia Diabetes Mellitus II Arthritis Hypertension
Charlson Co-Morbidity Index	27% of sample with estimated 10-year survival	
Ejection fraction %	37.7 (SD = 15.35)	
Disease trajectory	NYHA Class II—8% NYHA Class III—67% NYHA Class IV—25%	

**Table 3:** Dyad congruence questions and answers.

Question	HF	Caregiver
What are your HF goals?	Stay out of the hospital Exercise Diet Take mediation as prescribed	Stay out of the hospital Symptom Management Diet Take medication as prescribed Exercise
Do you think you are compliant with your medications? Is the patient compliant with his/her medications?	YES	YES
Do you think you are compliant with your exercise? Is the patient compliant with his/her exercise regimen?	YES	YES
Do you think you are compliant with your diet restrictions? Is the patient compliant with his/her diet restrictions?	YES	YES
Do you think you manage your heart failure symptoms? Does the HF patient manage his/her heart failure symptoms?	YES	YES
Since discharge, have caregiving tasks increased?	YES	YES
Have caregiving hours increased?	YES	YES
Has caregiving become burdensome?	YES	YES
What has been the most difficult since discharge? (Caregivers only)		Change in medications Additional outpatient appointments Adjusting to higher level of care

____ = Congruent; ____ = Semi-congruent.

**Table 4:** Zarit burden interview—frequency of response by question.

Question	Never	Rarely	Sometimes	Quite Frequently	Nearly Always
1	7	0	5	0	0
2	4	5	3	0	0
3	4	2	5	0	1
4	7	2	1	2	0
5	9	1	2	0	0
6	9	2	1	0	0
7	2	4	2	3	1
8	3	1	4	2	2
9	9	1	1	1	0
10	7	3	1	1	0
11	5	3	2	2	
12	8	2	1	1	0
13	9	1	1	1	0
14	4	2	2	3	1
15	5	3	1	0	3
16	8	3	1	0	0
17	8	3	1	0	0
18	7	3	2	0	0
19	9	2	1	0	0
20	8	1	2	1	0
21	7	1	3	1	0
22	7	1	4	0	0

**Table 5:** Patient and caregiver quality of life scores—SF-12.

	Physical Component Score	Mental Component Score
HF patients	32.24 (SD + 8.87)	53.85 (SD + 10.96)
Caregivers	47.52 (SD + 7.67)	52.8 (SD + 8.46)

## Data Availability

Data is not available to publish in public archives.

## Appendix A. The Zarit Burden Interview

0: never
1: rarely
2: sometimes
3: quite frequently
4: nearly always

Please circle the response that best describes how you feel.

**Question**	**Score**				
1 Do you feel that your relative asks for more help than he/she needs?	0	1	2	3	4
2 Do you feel that because of the time you spend with your relative, you don’t have enough time for yourself?	0	1	2	3	4
3 Do you feel stressed between caring for your relative and trying to meet other responsibilities for your family or work?	0	1	2	3	4
4 Do you feel embarrassed over your relative’s behavior?	0	1	2	3	4
5 Do you feel angry when you are around your relative?	0	1	2	3	4
6 Do you feel that your relative currently affects our relationships with other family members or friends in a negative way?	0	1	2	3	4
7 Are you afraid of what the future holds for your relative?	0	1	2	3	4
8 Do you feel your relative is dependent on you?	0	1	2	3	4
9 Do you feel strained when you are around your relative?	0	1	2	3	4
10 Do you feel your health has suffered because of your involvement with your relative?	0	1	2	3	4
11 Do you feel that you don’t have as much privacy as you would like because of your relative?	0	1	2	3	4
12 Do you feel that your social life has suffered because you are caring for your relative?	0	1	2	3	4
13 Do you feel uncomfortable having friends over because of your relative?	0	1	2	3	4
14 Do you feel that your relative seems to expect you to take care of him/her as if you were the only one he/she could depend on?	0	1	2	3	4
15 Do you feel that you don’t have enough money to take care of your relative in addition to the rest of your expenses?	0	1	2	3	4
16 Do you feel that you will be unable to take care of your relative much longer?	0	1	2	3	4
17 Do you feel you have lost control of your life since your relative’s illness?	0	1	2	3	4
18 Do you wish you could leave the care of your relative to someone else?	0	1	2	3	4
19 Do you feel uncertain about what to do about your relative?	0	1	2	3	4
20 Do you feel you should be doing more for your relative?	0	1	2	3	4
21 Do you feel you could do a better job caring for your relative?	0	1	2	3	4
22 Overall, how burdened do you feel caring for your relative?	0	1	2	3	4

Interpretation of scores are as follows:

0–21: little or no burden.
21–40: mild to moderate burden.
41–60: moderate to severe burden.
61–88: severe burden.
